# Extracting a biologically latent space of lung cancer epigenetics with variational autoencoders

**DOI:** 10.1186/s12859-019-3130-9

**Published:** 2019-11-25

**Authors:** Zhenxing Wang, Yadong Wang

**Affiliations:** School of Computer Science and Technology, Harbin Institute of Technology, Harbin, 150001 China

**Keywords:** DNA methylation, Lung cancer, Variational autoencoder

## Abstract

**Background:**

Lung cancer is one of the most malignant tumors, causing over 1,000,000 deaths each year worldwide. Deep learning has brought success in many domains in recent years. DNA methylation, an epigenetic factor, is used for model training in many studies. There is an opportunity for deep learning methods to analyze the lung cancer epigenetic data to determine their subtypes for appropriate treatment.

**Results:**

Here, we employ variational autoencoders (VAEs), an unsupervised deep learning framework, on 450K DNA methylation data of TCGA-LUAD and TCGA-LUSC to learn latent representations of the DNA methylation landscape. We extract a biologically relevant latent space of LUAD and LUSC samples. It is showed that the bivariate classifiers on the further compressed latent features could classify the subtypes accurately. Through clustering of methylation-based latent space features, we demonstrate that the VAEs can capture differential methylation patterns about subtypes of lung cancer.

**Conclusions:**

VAEs can distinguish the original subtypes from manually mixed methylation data frame with the encoded features of latent space. Further applications about VAEs should focus on fine-grained subtypes identification for precision medicine.

## Background

Lung cancer is one of the most malignant tumors with the fastest growth in morbidity and mortality, causing over 1,000,000 deaths each year. There are two common histological subtypes of lung cancer, lung adenocarcinoma (LUAD) and lung squamous cell carcinoma (LUSC). In order to understand the heterogeneity of lung cancer, many researchers have done a lot of work based on immune-response genes, DNA mutations and DNA methylation [1–4]. As a well-defined epigenetic factor, DNA methylation plays an important role in pathways as well as regulation of gene expression, so it can be used for monitoring of cancer diagnosis, development and treatment. However, with lung cancer rates progressively increasing, more efficient methods are needed for precision medicine finding ways to target subtypes for effective treatment.

In recent years, deep learning has been performed and achieved state-of-art performances in many domains, including speech, image classification, text and natural language processing, but has seen slow adoption for in bioinformatics[5]. Nevertheless, several studies have revealed interesting results by training deep models to diagnose melanoma based on image classification or to predict impact of non-coding variants [6, 7]. However, extracting specific biological features remains challenging.

Variational autoencoders (VAEs), which are unsupervised deep learning approaches, have become more and more popular in the research area. Interestingly, through feature compression and nonlinear activation functions, the VAEs can capture an underlying data manifold from input data [8]. Compared to traditional autoencoders, the VAEs are stochastic and learn to interpret the distribution of features over samples while the former are deterministic discriminative models and trained by minimizing the empirical reconstruction error [9]. There have been some successful efforts to apply VAEs to biological datasets. For instance, Way and Greene used RNA-seq data from TCGA as input to a VAE and obtained RNA-seq expression patterns in specific cancer-types [9]. Titus et al. learned a meaningful representation of the measured methylome for different subtypes of breast cancer by employing a VAE on 450K DNA methylation data [10].

Here, we use a VAE model in the study of lung cancer - including two subtypes: LUAD and LUSC - epigenetic data. Although the samples of lung cancer from TCGA are labelled, we chose unsupervised learning here instead of supervised learning to verify whether the reconstructed features by VAEs can represent the original data labels. We demonstrate that the encoded 100-dimensional latent space holds meaningful information of the original methylome. It is showed that the features of latent space represents the patterns of LUAD and LUSC epigenetics and the VAE model may be available for analysing DNA methylation data to extract features associated with subtypes.

## Results

In order to verify the feasibility of VAEs to extract a biologically meaningful latent space from DNA methylation data, we employed a VAE model on the top 300,000 probes that were chosen by median absolute deviation (MAD) of methylation beta values across 917 samples containing LUAD and LUSC subtypes. The 300,000 features were encoded to an intermediate layer with 100 dimensions which were then encoded back to 300,000 dimensions by a non-linear combination. For an insight into the 100 dimensions space, t-Distributed Stochastic Neighbor Embedding (t-SNE) [11] method was performed to reduce the dimensionality to 2. Then the 2D features were used to train logistic regression classifiers over the merged data frame.

### Latent features of the VAE model

**Fig. 1:**
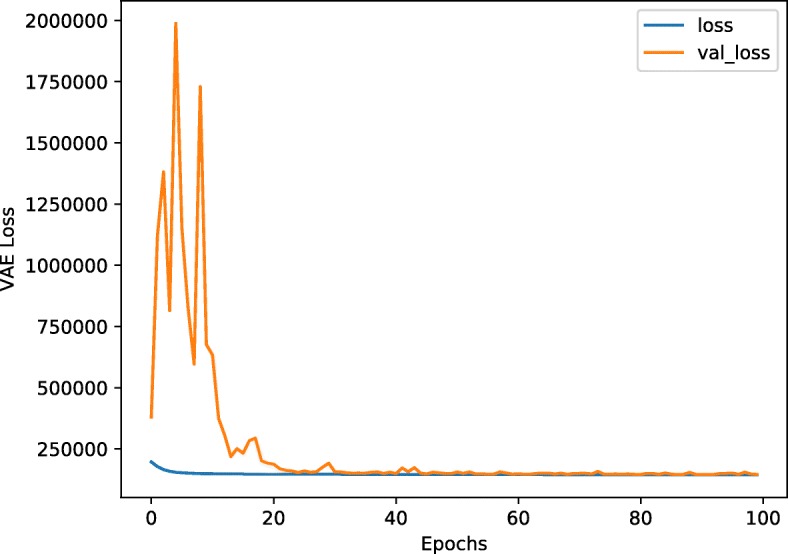
The process of the model training.The training loss is indicated by the blue line while the validation loss is indicated by the orange line during model training. The two loss values approach the same level after about 30 epochs

**Fig. 2:**
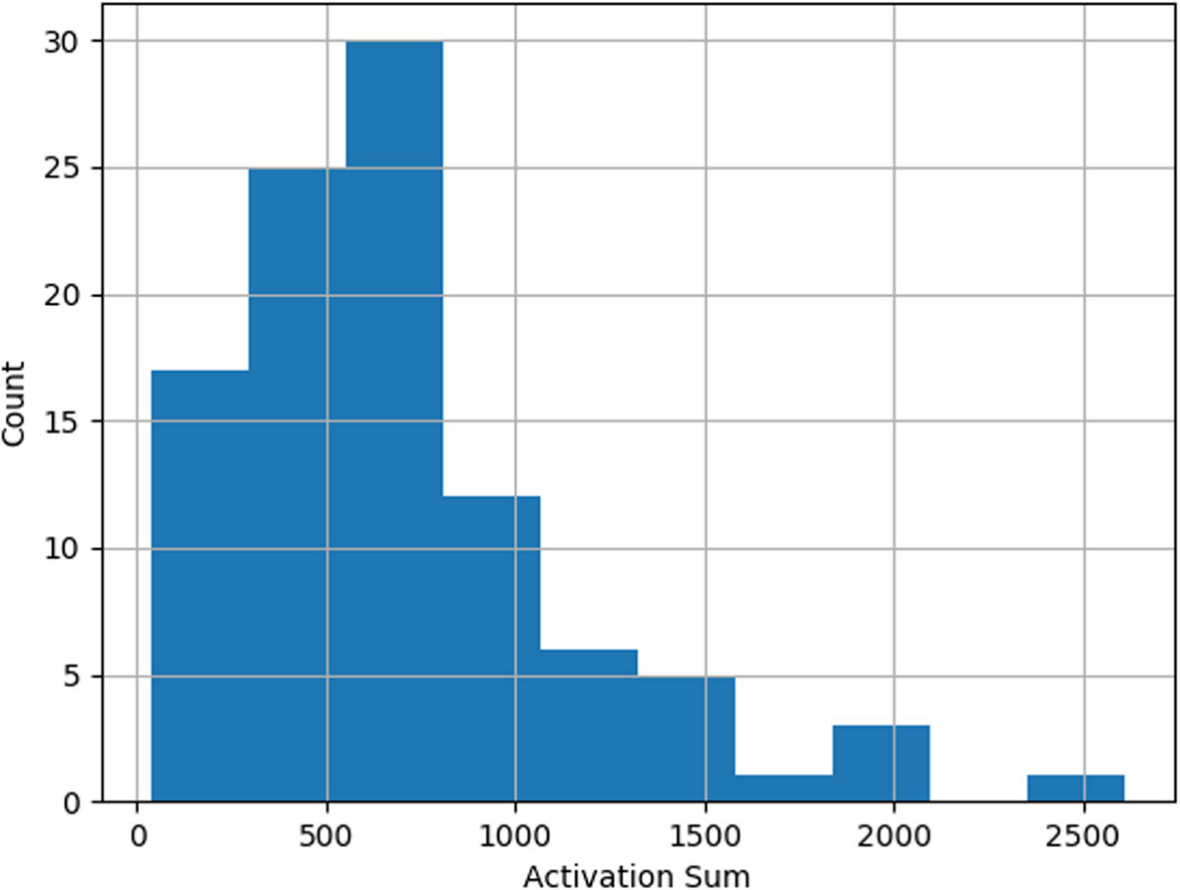
The histogram for the 100-dimensional features of the latent space. Most of the latent features are activated with sum >500

**Fig. 3:**
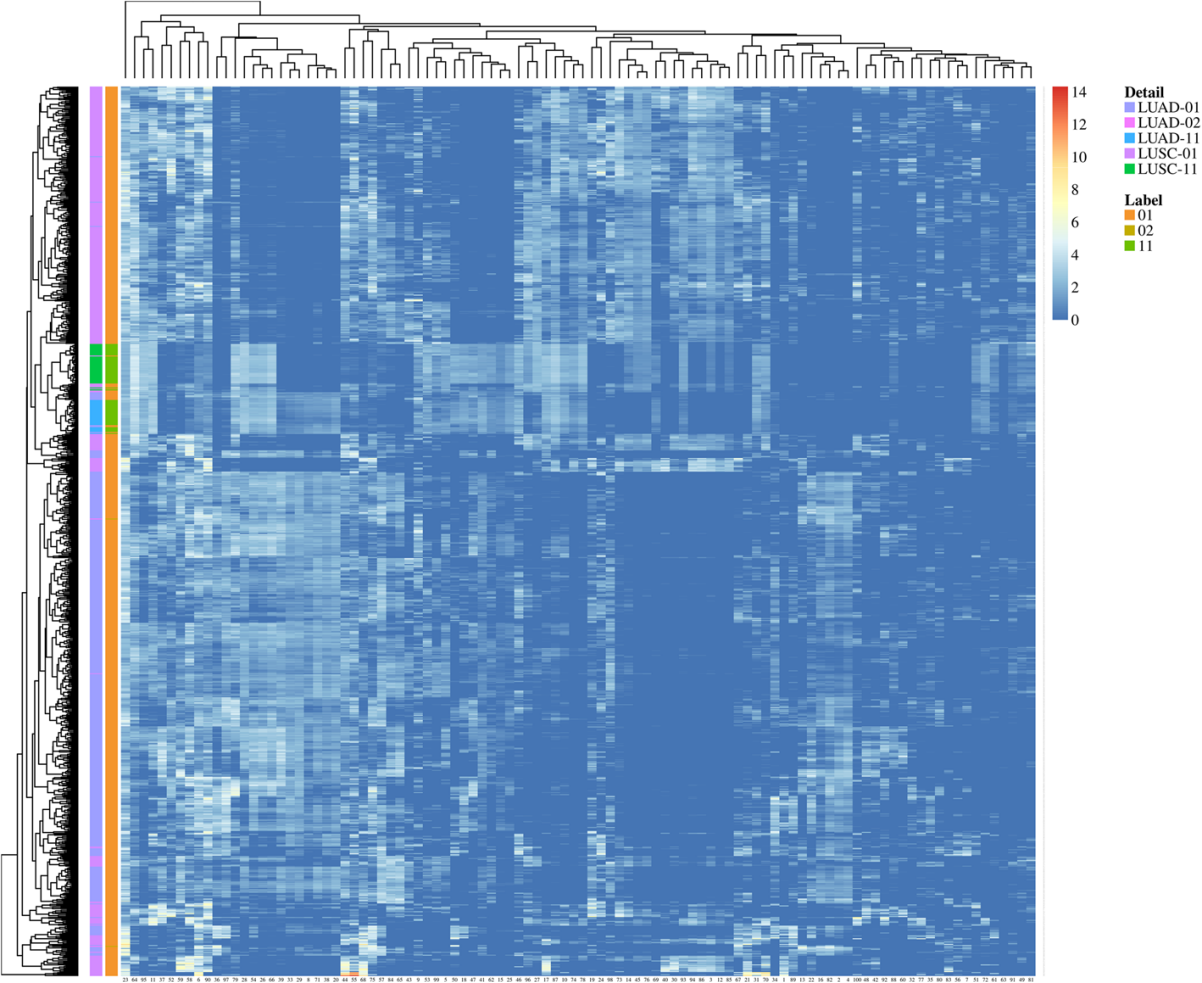
The heatmap of clustering results with the 100-dimensional latent features on 919 samples. Rows represent samples, which are annotated with “Detail” and “Label” color bars. For the “Label” bar, 01 or 02 represents tumor samples and 11 represents normal samples. Columns represent the latent features

It took about 2 hours to complete the model training on a server (Ubuntu 16.04.6) with 1T memory and no GPUs. The process of model training is shown in Fig. 1, where the validation loss drops rapidly after 10 epochs and then remains at a low level. The activation sum of most features in the latent space is high which indicates that the model is not zeroing out features (Fig. 2). Figure 3 shows the results of unsupervised hierarchical clustering with the 100-dimensional features of the latent space on the merged data frame. It can be seen that all samples are roughly grouped into four classes which are consistent with their original labels. We thought that the underlying DNA methylation patterns of different classes could be captured by the 100-dimensional features of the latent space. However, some LUSC-01 samples were classified into the LUAD-01 group, which indicated that a small fraction of LUSC tumor samples may have similar DNA methylation expression with the LUAD tumor. The distance between the two normal classes (LUAD-11 and LUSC-11, which gather in the middle part in Fig. 3) is smaller than that between the two tumor classes.

### Dimensionality reduction

In order to further investigate the relative information of the latent features, a well-known feature compress and visualization method, the t-SNE, was performed on the 100-dimensional features of the latent space resulting in 2D features.

**Fig. 4:**
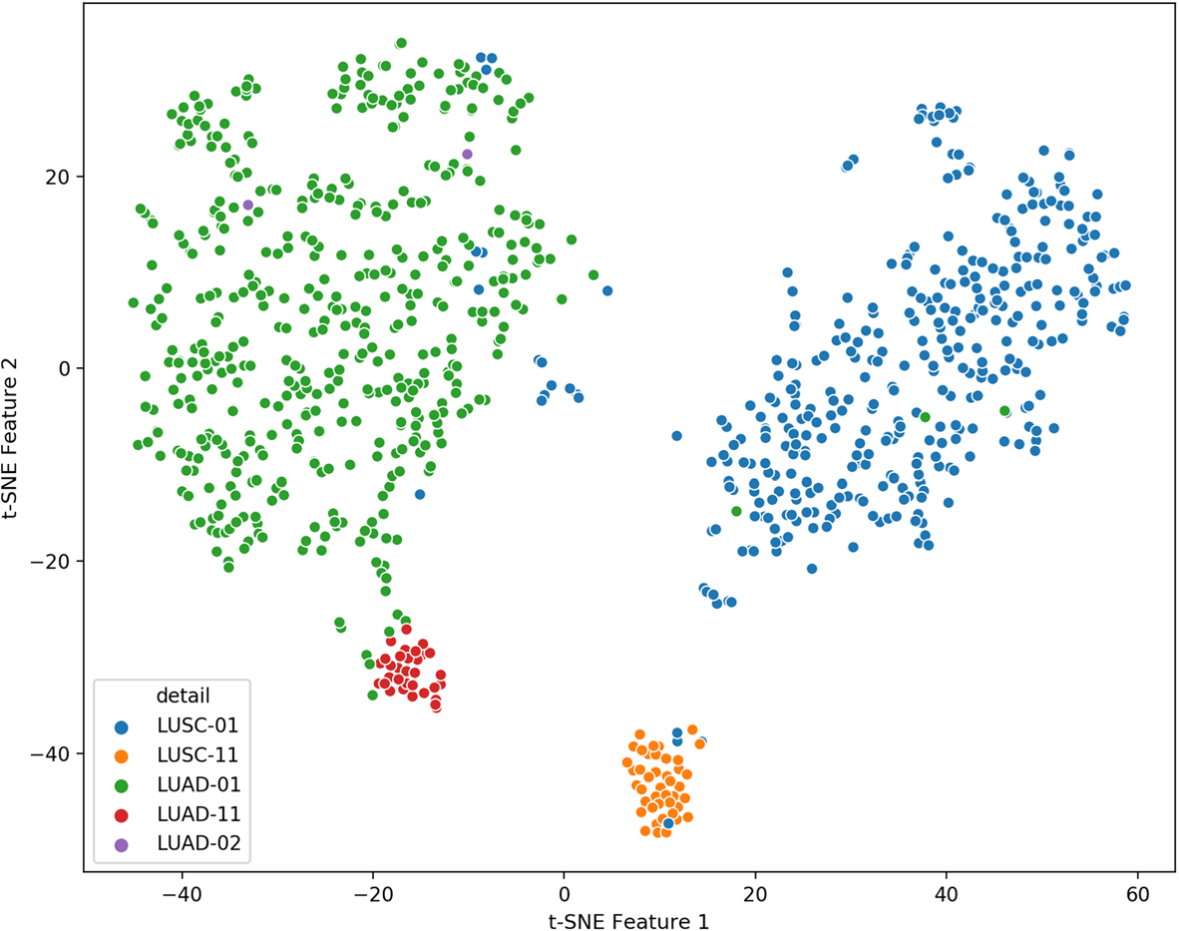
The scatter of 919 samples with the 2D t-SNE features. The x-axis represents the t-SNE feature 1, and the y-axis represents the t-SNE feature 2. Each point with a color represents a sample of the corresponding subtype, as the legend shows

A scatter for the 2D features was plotted which showed an obvious distribution with four main classes showing separation (Fig. 4). The distance among the four classes was captured significantly, revealing the underlying different DNA methylation patterns. It should be noted that several samples failed to fall in the expected area (for example, 3 LUAD-01 samples mixed into the area of LUSC-01 samples) suggesting that a small fraction of LUAD samples possess similar DNA methylation pattern with that of LUSC, and vice versa. This analysis is consistent with the result of Fig. 3.

**Fig. 5:**
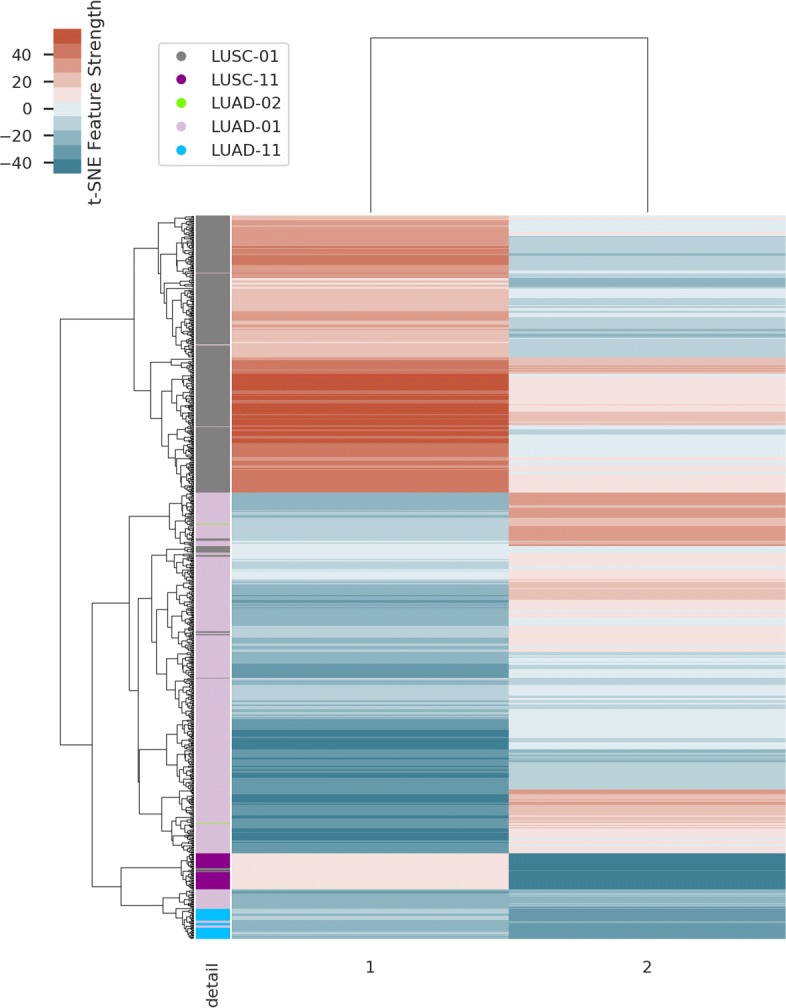
The heatmap of clustering results with the 2D compressed features on 919 samples associated with their original labels. Rows represent samples, which are annotated with the “detail” bar. Columns represent the 2D t-SNE features

Results of unsupervised hierarchical clustering with the 2D t-SNE features on the merged data frame are shown in Fig. 5, which are in accordance with Fig. 3. However, two normal classes gathered in the lower portion of Fig. 5, which demonstrated that the distance among the original four labelled subtypes was recalculated after dimensionality reduction.

### Classification with the t-SNE features

**Fig. 6:**
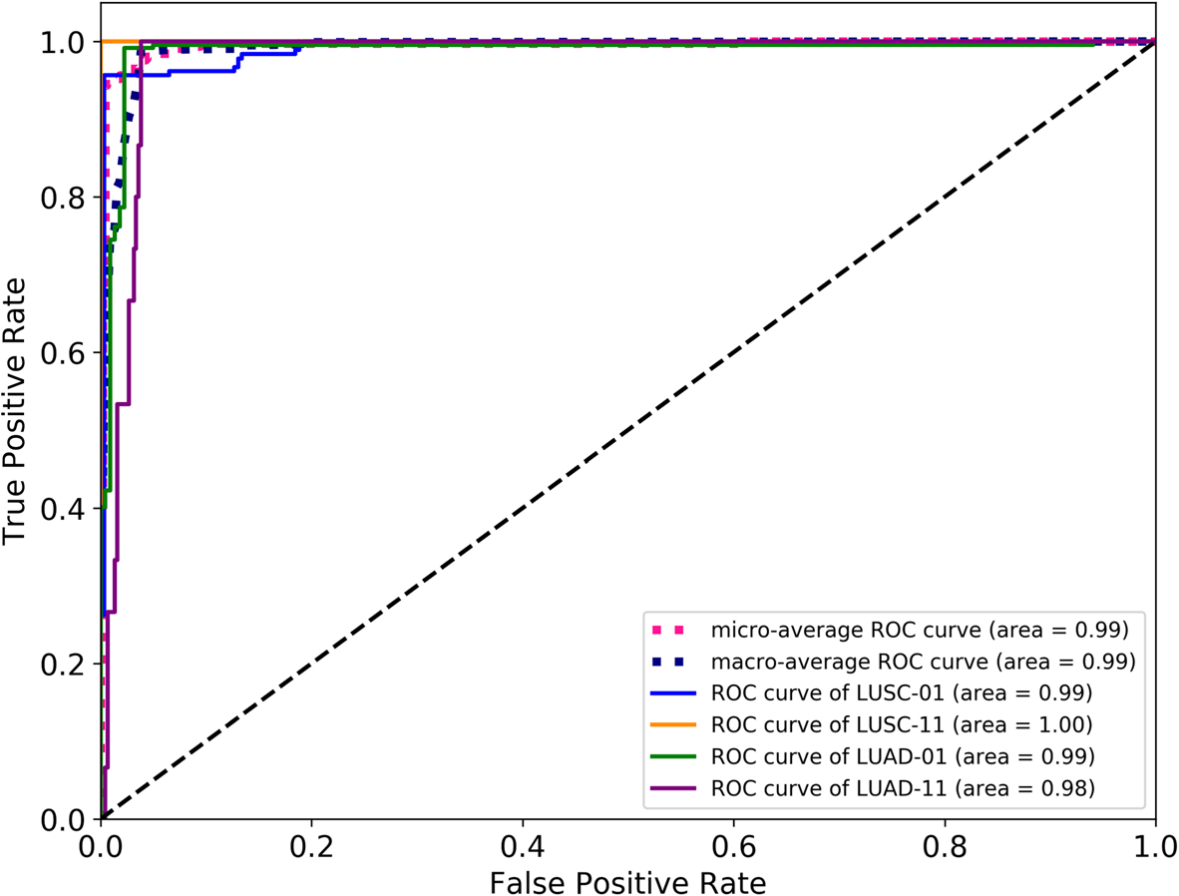
ROC curves of the four bivariate classifiers on test data. As the legend shows, the ROC curve for classifier of LUSC-01 is indicated by the blue line, and so on

To test the utility of the compressed 2D features, logistic regression classifiers were performed on the merged data frame with “1 vs The Rest” analyses by sklearn module [12]. A half of the samples were used to train the logistic regression model and the others were used to validate the performance of the model. The performance of classifiers are shown in Table 1. Classification precisions were obtained for four subtypes, equal to 0.92, 0.99, 0.75 and 1.00 respectively for LUAD-01, LUSC-01, LUAD-11 and LUSC-11 samples. There are two reasons leading to the lower precision for classifier of LUAD-11: i) the DNA methylation pattern of a small fraction of LUAD-01 samples overlapped with that of LUAD-11 (as shown in Fig. 4), and ii) the number of tested normal samples was small. The ROC curves of the four classifiers are shown in Fig. 6. The AUCs of four classifiers are all close to 1, and AUCs of micro-average and macro-average ROC curve are also high, suggesting that the classifiers consisting of the 2D t-SNE features can effectively classify the four clusters.

**Table 1:** Performance table of the logistic regression classification on test dataset based on 2D t-SNE features

	#Samples	Recall	F1-score	Precision
LUAD-01	239	0.99	0.96	0.92
LUSC-01	183	0.96	0.97	0.99
LUAD-11	15	0.20	0.32	0.75
LUSC-11	22	1.00	1.00	1.00
micro average	459	0.95	0.95	0.95
macro average	459	0.79	0.81	0.92

## Discussion

We transfer the application of *Tybalt*, which was developed to learn a latent space on pan-cancer RNA-seq data by Way and Greene, on epigenetic data from lung cancer to extract a meaningful relevant space. The above analyses show that epigenetic data of lung cancer is suitable for unsupervised deep learning to mine its subtypes. Moreover, it shows that the VAE model could extract a biologically relevant space and the meaningful information still can be captured by the further compressed features after dimensionality reduction with the t-SNE method.

The successful applications of deep learning on many domains give us a clue that it should be used for precision medicine for more effective treatments in the context of cancer rates and cancer-related mortality fast increasing.

With the data of TCGA released publicly, large-scale dataset and pan-cancer analyses can be achieved by deep learning methods. In the future work, pan-cancer 450K DNA methylation data can be trained by the VAEs to get a latent representative space relevant to large-scale datasets from multiple tumors. To better understand the tumors, further analyses and multi-omic data should be involved, for example, fine-grained subtypes of a specific tumor should be identified, and gene ontology (GO) enrichment analyses can be run on CpGs with high weights, and non-coding RNA data can be added into input [13, 13–18].

## Conclusions

In this work, we demonstrate that the epigenetic data of lung cancer samples is capable of unsupervised deep learning with VAEs. A biologically meaningful latent space can be extracted by the VAE model from the manually merged dataset, which represents the distribution about different subtypes of samples credibly. By comparing the results of unsupervised hierarchical clustering with the original labels of samples, VAEs can capture the different methylation expression patterns for various subtypes.

## Methods

### Data

All level-3 plain files of Illumina HumanMethylation450 (450K) DNA methylation data for LUAD and LUSC samples were downloaded from the The Cancer Genome Atlas (TCGA) project (https://portal.gdc.cancer.gov/) through the GDC data transfer tool.

**TCGA-LUAD dataset** contains 507 LUAD samples. It consists of 32 tumor-adjacent normal samples (short for LUAD-11) and 475 tumor samples. For tumor samples, there are 473 primary solid tumor samples (short for LUAD-01) and 2 recurrent solid tumor samples (short for LUAD-02).

**TCGA-LUSC dataset** contains 412 LUSC samples. It consists of 42 tumor-adjacent normal samples (short for LUSC-11) and 370 solid tumor samples (short for LUSC-01).

Table 2 shows the summary of the two datasets. All the 919 files were merged into one big data frame with Pandas [19] and Numpy [20] modules in Python language. In filtering steps, we removed the probes that were SNP-associated and sex-specific and contained any NA beta-value, resulting in a data frame with a dimension of 356,464 * 919.

**Table 2:** Summary of 450K DNA methylation datasets for TCGA-LUAD and TCGA-LUSC

Subtype	LUAD			LUSC	
Tissue	Tumor		Normal	Tumor	Normal
n	473	2	32	370	42
Code	LUAD-01	LUAD-02	LUAD-11	LUSC-01	LUSC-11

### Model summary

We extend *Tybalt* [9], a VAE model, to extract a biological relevant space for lung cancer epigenetic data in this work. The original *Tybalt* was developed for extract a relevant latent space from cancer transcriptomes of 10,459 tumors. The original model consisted of an encoder and a decoder where 5,000 input selected genes were encoded to 100 latent features and reconstructed back to the 5,000 genes. Way et al. chose Keras (version 2.2.2) [21] to build the model with a TensorFlow backend (version 1.5.0) [22] and trained it with an Adam optimizer, included batch normalization in the encoder and sigmoid activation in the decoder. In our work, the dimension of the input data was 300,000 * 919 of which the 300,000 CpGs were selected by median absolute deviation (MAD) over our merged data frame. We selected the parameters in *Tybalt* with the following values: 50 for batch size, 0.0005 for learning rate, 1 for *κ*, 90/10 for training/validation ratio. We changed the epochs from 50 to 100, expecting better training.

### Latent features

The latent space consisted of 100 features compressed from 300,000 CpGs. First, the 100-dimensional latent features were evaluated whether they may represent the different methylation patterns for LUAD/LUSC subtypes. So, we performed unsupervised hierarchical clustering on the merged data frame with the 100-dimensional latent features then compared the clustering results with the original labels of each sample.

### Dimensionality reduction

For a more intuitive representation, we performed dimensionality reduction on the 100-dimensional latent features. The t-SNE method converts the Euclidean distance to probability distribution using Gaussian distribution making it suitable for feature compression and visualization [11]. We performed the t-SNE method on the 100-dimensional latent features resulting in a 2D features. Then we performed the unsupervised hierarchical clustering once more on our merged data frame with the 2D features and compared the clustering results with the original labels of each sample.

### Classification with the 2D t-SNE features

In order to test the utility of the 2D t-SNE features, “1 vs The Rest” logistic regression classifiers were trained with the 2D features from t-SNE analyses. Specially, to simplify the model training, the labels of the only 2 LUAD-02 samples were converted to “LUAD-01”. So, four bivariate classifiers (respectively for LUAD-01, LUSC-01, LUAD-11 and LUSC-11) were developed to classify samples using the 2D features. The merged data frame was randomly split into 50/50 using Pandas module for training/testing sets where 50% of the samples from each subtype were included.

## Abbreviations

450K: Illumina HumanMethylation450
GO: Gene ontology
LUAD: Lung adenocarcinoma
LUSC: Lung squamous cell carcinoma
MAD: Median absolute deviation
t-SNE: t-Distributed stochastic neighbor embedding
TCGA: The cancer genome atlas
VAEs: Variational autoencoders
